# Allan-Herndon-Dudley syndrome associated with *SLC16A2* c.1384G>A (p.Gly462Arg): A family case report with retrospective molecular diagnosis in a deceased sibling

**DOI:** 10.1097/MD.0000000000051483

**Published:** 2026-11-06

**Authors:** Liwei Li, Jiarui Li, Zhenyan Li, Huijing Lv, Jian Sun, Xiufeng Wang, Shirui Hao, Lina Zheng, Shuping Wang, Zhenpu Li, Shuping Li

**Affiliations:** a Department of Clinical Laboratory, Xingtai People’s Hospital, Xingtai, China; b Department of Medical Imaging Technology, School of Medical Technology, Tianjin Medical University, 23rd Imaging Class 2, Tianjin, China; c Department of Clinical Laboratory, Xingtai Women and Children’s Health Hospital, Xingtai, China; d Department of Clinical Laboratory, Hebei Eye Hospital, Xingtai, China; e Department of Pediatrics, Xingtai People’s Hospital, Xingtai, China; f Department of Clinical Laboratory, Peking University First Hospital Ningxia Women and Children’s Hospital (Ningxia Hui Autonomous Region Maternal and Child Health Hospital), Yinchuan, China; g Department of Operating Room, Xingtai People’s Hospital, Xingtai, China.

**Keywords:** Allan-Herndon-Dudley syndrome, neonatal dried blood spot, p.Gly462Arg, retrospective molecular diagnosis, *SLC16A2*

## Abstract

**Rationale:**

Allan-Herndon-Dudley syndrome (monocarboxylate transporter 8 deficiency) is an X-linked disorder caused by pathogenic variants in *SLC16A2*, with severe neurodevelopmental impairment and a characteristic high-triiodothyronine (T3)/low-thyroxine (T4) thyroid profile. *SLC16A2* c.1384G> A (p.Gly462Arg; NM_006517.5) was reported previously. We describe a Chinese family with the same variant, including retrospective molecular confirmation in a deceased maternal half brother.

**Patient concerns:**

The proband was evaluated at 4.3 years for profound developmental delay. He could not sit, stand, or walk independently and had an intelligence quotient of 36, axial hypotonia with peripheral hypertonia, dystonia, muscle atrophy, joint contractures, and bilateral cryptorchidism.

**Diagnosis:**

Thyroid testing showed elevated free triiodothyronine and total triiodothyronine, decreased free thyroxine and total thyroxine, and mildly elevated thyroid-stimulating hormone. Brain magnetic resonance imaging showed delayed myelination, a thin corpus callosum, and widened frontal subarachnoid spaces. Whole-exome sequencing identified hemizygous *SLC16A2* c.1384G> A (p.Gly462Arg); Sanger sequencing confirmed the variant in the proband and heterozygosity in his mother. The same hemizygous variant was detected in DNA from the archived neonatal dried blood spot of a maternal half brother who had severe developmental delay and died at 16 months. Thyroid tests, neuroimaging, and cause of death were not recorded for the deceased child.

**Interventions:**

The proband received physical, occupational, and speech therapy and underwent bilateral orchiopexy. Triiodothyroacetic acid was not available at diagnosis.

**Outcomes:**

At the last documented follow-up at age 5 years, he remained unable to sit, stand, or walk independently. Clinical notes described improved head control, but no repeat standardized developmental score was available. Epileptiform electroencephalogram discharges persisted, but no clinical seizures were documented.

**Lessons:**

This family provides additional clinical and segregation evidence for p.Gly462Arg. Testing an archived neonatal blood spot established the diagnosis in the deceased sibling and strengthened genetic counseling. *SLC16A2* testing should be considered in boys with unexplained severe neurodevelopmental delay and a high-triiodothyronine/low-thyroxine pattern. Functional studies are still required to define the effect of p.Gly462Arg on monocarboxylate transporter 8 trafficking and thyroid hormone transport.

## 1. Introduction

Allan-Herndon-Dudley syndrome (AHDS), also termed monocarboxylate transporter 8 (MCT8) deficiency, is an X-linked disorder caused by pathogenic variants in *SLC16A2*. MCT8 is a highly specific thyroid hormone transporter that is important for thyroid hormone entry into the developing brain and for systemic thyroid hormone homeostasis. Loss of MCT8 function produces a characteristic combination of cerebral thyroid hormone deprivation and peripheral thyrotoxicosis, with severe neurodevelopmental impairment, abnormal muscle tone and movement, impaired growth, and a biochemical profile typically characterized by elevated T3 with low or low-normal T4 and normal or mildly increased thyroid-stimulating hormone (TSH).^[1–4]^

The clinical and molecular spectrum of MCT8 deficiency has expanded substantially. An international natural history study of 151 affected individuals identified 73 distinct MCT8 variants,^[2]^ and a 2025 integrative analysis assembled data from 371 patients carrying 155 different *SLC16A2* variants, including 63 missense variants.^[3]^ These data demonstrate meaningful genotype–phenotype relationships while also showing that clinical severity is more closely related to residual transporter function than to transmembrane location alone.^[3]^ Current UniProt topology annotation places Gly462 within MCT8 transmembrane helix 11 (residues 448–468).^[5]^

Despite advances in molecular diagnosis, delayed recognition remains common because the early clinical phenotype can be nonspecific, and T3 is not always included in routine thyroid testing.^[4]^ A 2017 report described an unrelated Spanish boy with the same nucleotide substitution, c.1384G> A.^[6]^ The original article rendered the protein consequence inconsistently, but using the current reference transcript NM_006517.5, c.1384G> A corresponds to p.Gly462Arg. Accordingly, the present study does not claim novelty of the variant. Instead, it contributes detailed clinical and biochemical characterization of a Chinese proband, intrafamilial segregation in 2 affected maternal half brothers and their mother, and retrospective molecular diagnosis of the deceased half-brother using an archived neonatal dried blood spot. Archived neonatal dried blood spots can provide DNA suitable for genomic analysis even after prolonged storage.^[7]^

## 2. Case presentation

The proband was born at term after an uneventful pregnancy and delivery to nonconsanguineous, healthy Chinese parents, with normal Apgar scores. Developmental delay became evident in early infancy. He had no sustained head control at 3 months, was unable to roll over at 4 months, and never achieved independent sitting, standing, or walking. Speech remained limited to babbling, and visual tracking was poor. At 4.3 years, his weight was 12 kg (<3rd percentile) and height was 102 cm (<2nd percentile). A contemporaneous head circumference measurement was not available in the records; therefore, microcephaly could not be objectively confirmed.

At 4.3 years, neurological examination showed a characteristic facial appearance with large ears, an open mouth, and a “puppet-like” smile, axial hypotonia, peripheral hypertonia, dystonic movements, limb tremor, joint contractures, bilateral Babinski signs, and generalized muscle atrophy (Fig. 1A). Bilateral cryptorchidism was also present. Cognitive assessment documented severe intellectual disability with an intelligence quotient of 36. Electroencephalogram (EEG) demonstrated epileptiform discharges in the central frontal region, but no clinical seizures had been observed. Thyroid function testing showed free triiodothyronine (FT3) 6.35 pmol/L (reference 2.63–5.70), free thyroxine (FT4) 6.01 pmol/L (9.01–19.03), total T3 4.70 nmol/L (1.07–2.78), total T4 40.60 nmol/L (65.9–135.2), and TSH 6.83 mIU/L (0.35–4.94), consistent with the characteristic biochemical profile of MCT8 deficiency (Table S1, Supplemental Digital Content 1). Brain magnetic resonance imaging at 4.3 years showed delayed myelination estimated at approximately 11 months relative to age expectations, a thin corpus callosum, and widened frontal subarachnoid spaces (Fig. 1B). Conventional karyotyping showed 46,XY (Fig. 2A).

**Figure 1.**
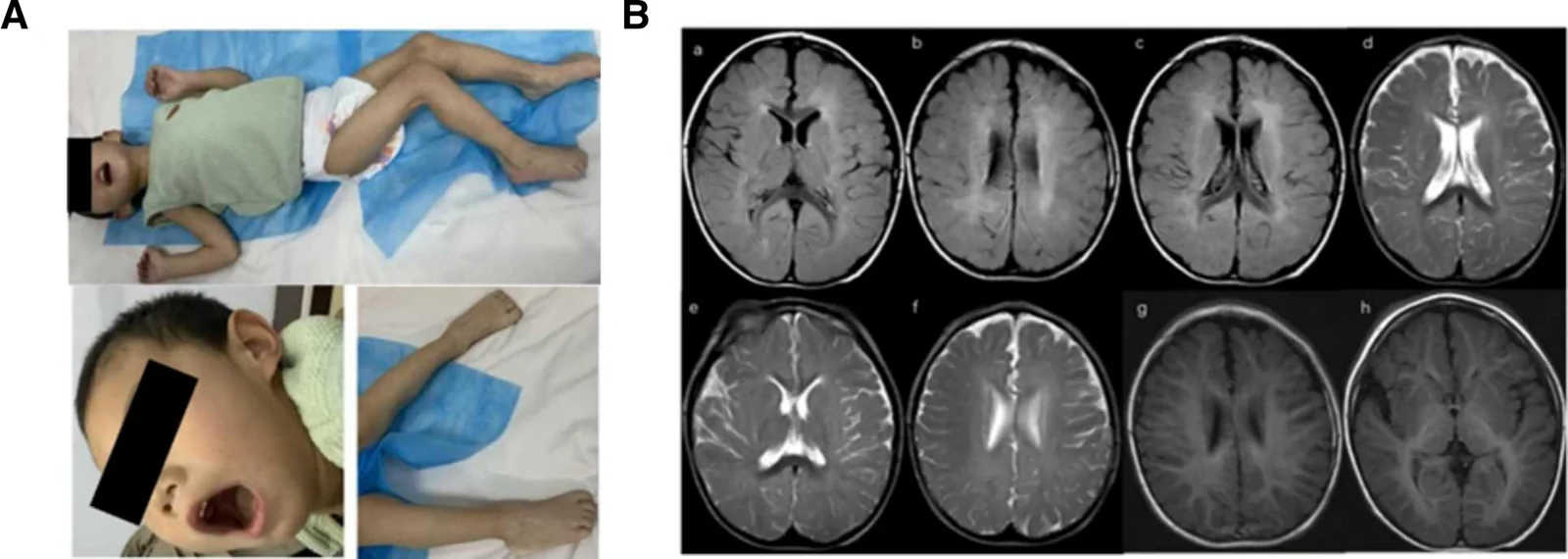
Clinical and magnetic resonance imaging findings in the proband. (A) Photographs at 4.3 years showing muscle wasting, contractures, and the facial appearance; the eyes are masked. (B) Axial magnetic resonance imaging at 4.3 years showing delayed myelination, a thin corpus callosum, and widened frontal subarachnoid spaces.

**Figure 2.**
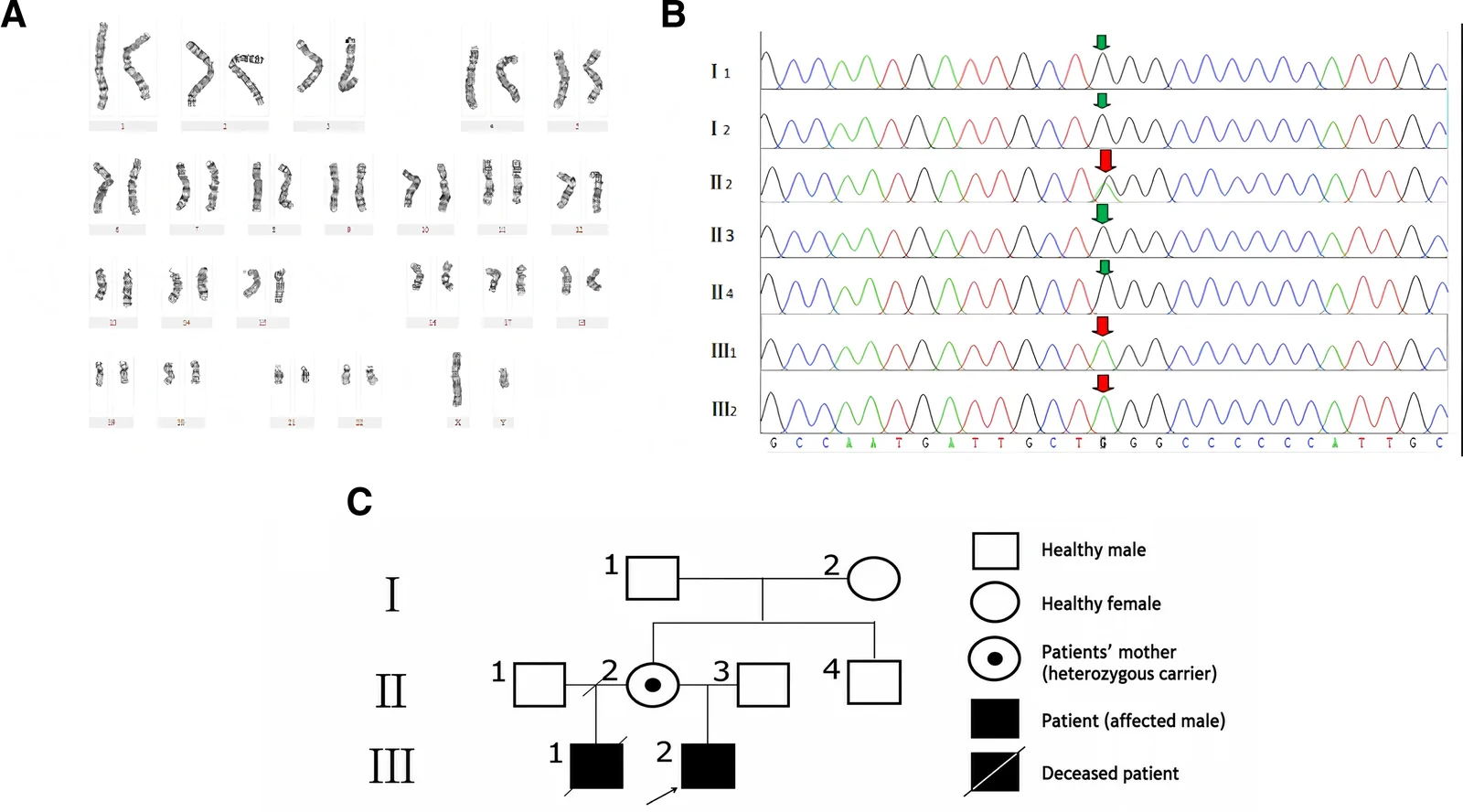
Karyotype, Sanger chromatograms, and pedigree. (A) Conventional karyotype of the proband: 46,XY. (B) Sanger traces at *SLC16A2* c.1384. The maternal grandparents (I-1, I-2), the proband’s father (II-3), and the maternal uncle (II-4) carried the reference allele. The mother (II-2) was heterozygous for c.1384G > A. The deceased maternal half brother (III-1) and the proband (III-2) were hemizygous. The deceased half brother’s father (II-1) was not tested. Green arrows mark the reference allele and red arrows the variant. (C) Pedigree. Open square: unaffected male; open circle: unaffected female; dotted circle: heterozygous carrier; filled square: affected male; slashed filled square: deceased affected male; arrow: proband.

Whole-exome sequencing (WES) was performed on genomic DNA from the proband in a clinical diagnostic setting and identified the hemizygous *SLC16A2* variant NM_006517.5:c.1384G> A, predicting p.Gly462Arg in exon 5. The variant was absent from the population databases queried in the original analysis, including gnomAD, ExAC, and the 1000 Genomes Project, and from the local East-Asian exome dataset used by the testing laboratory. The original WES was performed using a capture kit from Beijing Genomics Institute, followed by high-throughput sequencing on the DNBSEQ-T7 platform. The mean target depth was 134.16×, with 99.91% target-region coverage; 99.39% and 98.26% of target bases were covered at ≥10× and ≥20×, respectively. Sanger sequencing confirmed the variant in the proband and demonstrated heterozygosity in his mother (Fig. 2B). The mother had mildly elevated total T3 (2.91 nmol/L; reference 1.07–2.78), with FT3 at the upper end of the reference interval, FT4 at the lower end of the reference interval, and normal TSH (Table S1, Supplemental Digital Content 1); no major motor or intellectual impairment was documented.

Family history review identified another son of the mother from a previous marriage, making him the proband’s maternal half-brother. This child also had a normal Apgar score at birth but developed marked growth and developmental delay and was described in the available records as having cerebral palsy and profound intellectual disability by 11 months. He died at 16 months. The surviving records did not provide quantitative anthropometric measurements, detailed developmental milestones, a formal neurological examination, thyroid function tests, brain magnetic resonance imaging or EEG findings, treatment history, or a documented cause of death. A preserved neonatal dried blood spot was available. Sanger sequencing using DNA obtained from this archived sample identified the same hemizygous c.1384G> A (p.Gly462Arg) variant, providing retrospective molecular confirmation of AHDS in the deceased child. Segregation testing shown in Figure 2B demonstrated the reference sequence in the maternal grandparents (I-1 and I-2), the proband’s father (II-3), and the maternal uncle (II-4); the mother (II-2) was heterozygous, and both affected boys (III-1 and III-2) were hemizygous for c.1384G> A. The family pedigree is shown in Figure 2C. The deceased half-brother’s father (II-1) was not tested. The current family and the previously reported patient with the same nucleotide substitution are compared in Table 1.

**Table 1:** Clinical comparison of *SLC16A2* c.1384G > A (p.Gly462Arg) cases.

Case/source	Ancestry; age	Neurodevelopmental/neurological phenotype	Thyroid profile	Neuroimaging	Molecular/family and outcome
Rego et al, 2017**^[6]^**	Spanish; 10 yr at report (diagnosed at 7 yr)	Severe intellectual and motor disability; unable to speak or walk; axial hypotonia, limb hypertonia/spasticity, dystonic/dyskinetic movements, muscle atrophy	High free triiodothyronine, low free thyroxine, normal thyroid-stimulating hormone	Generalized delayed cerebral/cerebellar myelination; findings compatible with loss of brain volume	Hemizygous c.1384G> A; mother carrier; neurological worsening during follow-up
Present proband (III-2)	Chinese; 4.3 yr at evaluation; 5 yr at last follow-up	No independent sitting, standing, or walking; speech limited to babbling; intelligence quotient 36; axial hypotonia, peripheral hypertonia, dystonia, tremor, contractures, muscle atrophy	High free triiodothyronine /total triiodothyronine, low free thyroxine /total total thyroxine, mildly high thyroid-stimulating hormone	Delayed myelination, thin corpus callosum, widened frontal subarachnoid spaces	Hemizygous c.1384G>A; mother heterozygous; still nonambulatory at age 5; developmental scores not repeated
Present deceased maternal half-brother (III-1)	Chinese; died at 16 mo	Marked growth and developmental delay; cerebral palsy and profound intellectual disability by 11 mo; detailed milestones and examination not recorded	Not recorded	Not recorded	Same hemizygous c.1384G>A from stored neonatal dried blood spot; cause of death not recorded

Protein numbering follows NM_006517.5. “Not recorded” indicates that the information was absent from the available source records.

Variant interpretation was reassessed using the ACMG/AMP framework together with calibrated computational evidence. The variant was absent from the population datasets queried (PM2_Supporting), the REVEL score of 0.932 reached a published threshold for strong computational evidence (PP3_Strong), the proband’s phenotype and thyroid profile were highly specific for MCT8 deficiency (PP4), and the same hemizygous variant was identified in the similarly affected maternal half-brother with maternal heterozygosity (PP1). The prior unrelated case with c.1384G> A provides additional case-level support but was not independently scored in order to avoid overinterpretation. On this basis, the variant was classified as likely pathogenic (Table S2, Supplemental Digital Content 2).^[8,9]^ Gly462 lies within transmembrane helix 11 according to current UniProt annotation^[5]^; because no direct functional study of p.Gly462Arg was performed, this topological information was treated as biological context rather than as independent proof of pathogenicity.

Management was supportive. The proband received physical and occupational therapy for motor impairment, hypotonia, and contractures, speech therapy for communication difficulties, and bilateral orchiopexy for cryptorchidism. The available records confirm that these therapies were provided but do not reliably document their exact initiation dates, session frequency, or cumulative duration; therefore, these details are reported as unavailable rather than inferred. No disease-specific thyroid hormone-modulating treatment was initiated because triiodothyroacetic acid (Triac) was not available locally at the time of diagnosis (2019–2020). Follow-up assessments were performed at approximately 6-month intervals and included clinical growth, neurodevelopmental status, thyroid function, and seizure surveillance. At the last documented follow-up at age 5 years, the child remained unable to sit, stand, or walk independently. Clinical notes and caregiver report described improved head control, but no repeat standardized intelligence quotient, developmental quotient, Gross Motor Function Measure, or equivalent objective longitudinal scale was available. No clinical seizures were documented despite persistent epileptiform EEG discharges.

## 3. Discussion

We describe 2 maternal half brothers with a severe AHDS phenotype who share the hemizygous *SLC16A2* c.1384G>A (p.Gly462Arg) variant inherited through their heterozygous mother. A 2017 publication reported the same c.1384G> A nucleotide substitution in an unrelated Spanish boy with severe MCT8 deficiency.^[6]^ Thus, the principal contribution of the present report is not variant novelty, but independent familial evidence, detailed characterization in a Chinese family, and retrospective molecular confirmation in a deceased sibling using an archived neonatal dried blood spot.

MCT8 transports thyroid hormones across cell membranes and is particularly important for thyroid hormone availability in the developing central nervous system.^[1,4]^ Deficiency results in inadequate cerebral thyroid hormone action together with abnormal peripheral thyroid hormone metabolism, which helps explain the combination of severe neurological impairment and elevated circulating T3.^[2,4]^ The proband’s low weight, muscle atrophy, and abnormal thyroid profile are consistent with the peripheral hypermetabolic phenotype described in larger MCT8 cohorts.^[2,3]^ Although epileptiform EEG activity was present, no clinical seizures were documented during the available follow-up.

The p.Gly462Arg substitution affects a conserved residue within transmembrane helix 11 of MCT8 (residues 448–468 in UniProt P36021).^[5]^ The 2017 case with the same c.1384G> A substitution had severe cognitive and motor disability, inability to speak or walk, axial hypotonia with limb hypertonia and dystonic movements, delayed myelination, high FT3, and low FT4.^[6]^ These findings are broadly concordant with the present proband, although the available evidence does not support describing the phenotypes or complete thyroid profiles as identical. Large-scale data also demonstrate substantial phenotypic heterogeneity among *SLC16A2* missense variants.^[3]^ Table 1 therefore provides a direct clinical comparison of the previously reported c.1384G> A case with the 2 affected members of the current family.

The mother showed a mild biochemical carrier phenotype, with slightly elevated total T3 and otherwise near-normal thyroid indices. Heterozygous females are often less severely affected than hemizygous males, although biochemical and neurocognitive manifestations may occur, particularly in the setting of skewed X-chromosome inactivation.^[10]^ The mother’s findings are therefore compatible with carrier status but are not, by themselves, diagnostic evidence.

Treatment in the present case consisted of supportive multidisciplinary care. Triac is a thyroid hormone analogue that can enter cells independently of MCT8. In an international open-label phase 2 trial, Triac reduced serum T3 and improved several markers of peripheral thyrotoxicosis; neurological outcomes were exploratory and variable rather than demonstrating a uniform neurological response.^[11]^ A subsequent real-world cohort supported sustained biochemical and peripheral clinical benefits during longer-term treatment.^[12]^ Early diagnosis remains clinically relevant because it facilitates timely supportive management, genetic counseling, and consideration of disease-directed therapies where available or within appropriate specialist programs.^[5,11,12]^ Experimental approaches aimed at correcting central nervous system thyroid hormone deprivation remain under investigation and should not be presented as established clinical interventions.

The retrospective molecular diagnosis in the deceased half brother is a major strength of this report. Archived neonatal dried blood spots can preserve DNA suitable for genetic analysis and may allow molecular clarification when conventional testing was not performed during life.^[7]^ In this family, that result converted a historical clinical suspicion into a molecularly supported familial diagnosis and strengthened recurrence-risk counseling. The case also illustrates why MCT8 deficiency should be considered early in boys with severe unexplained neurodevelopmental delay, abnormal tone or movement, poor growth, and the characteristic high-T3/low-T4 thyroid profile.^[2,5]^

## 4. Limitations

This report has several limitations. First, no in vitro transport, trafficking, or expression study was performed for p.Gly462Arg; consequently, the molecular mechanism and degree of residual MCT8 function remain unknown. Second, although the WES capture-kit manufacturer, sequencing platform, mean target depth, and coverage metrics were available, the specific lot number of the Beijing Genomics Institute capture kit could not be retrieved from the retrospective laboratory records. Third, the deceased half brother’s clinical records were incomplete, including absence of objective head circumference, thyroid function, neuroimaging, detailed neurological findings, treatment history, and documented cause of death. Fourth, the supportive therapies received by the proband were not documented with sufficient detail to report exact initiation dates, frequency, or cumulative duration. Finally, longitudinal neurological follow-up did not include repeat standardized developmental or motor scales, so treatment-related neurological change cannot be quantified. Although c.1384G>A has been reported previously,^[6]^ independent functional validation of p.Gly462Arg remains unavailable.

## 5. Conclusion

This family report provides additional clinical and segregation evidence for the *SLC16A2* c.1384G> A (p.Gly462Arg) variant in a boy with a classic severe MCT8-deficiency phenotype, his heterozygous mother, and a similarly affected maternal half brother. The same nucleotide substitution has been reported previously in an unrelated patient^[6]^; therefore, the original contribution of this study lies in intrafamilial segregation and retrospective molecular diagnosis of the deceased sibling using an archived neonatal dried blood spot. Clinically, *SLC16A2* testing should be considered early in boys with unexplained severe neurodevelopmental delay, abnormal muscle tone or movement, and the characteristic thyroid hormone pattern of high T3 with low or low-normal T4. Future studies should directly test the effect of p.Gly462Arg on MCT8 expression, trafficking, and thyroid hormone transport, further define genotype–phenotype relationships, and evaluate additional patients with this specific variant.

## Author contributions

**Data curation:** Liwei Li, Jiarui Li, Zhenyan Li, Huijing Lv, Jian Sun, Xiufeng Wang, Shirui Hao, Lina Zheng, Shuping Wang, Zhenpu Li, Shuping Li.

**Formal analysis:** Liwei Li, Jiarui Li, Zhenyan Li, Huijing Lv, Jian Sun, Xiufeng Wang, Shirui Hao, Lina Zheng, Shuping Wang, Zhenpu Li, Shuping Li.




