# Supplementary material for: Allan-Herndon-Dudley syndrome associated with SLC16A2 c.1384G>A (p.Gly462Arg): A family case report with retrospective molecular diagnosis in a deceased sibling
Source: Medicine (Baltimore). 2026 Nov 6;105(45):e51483. doi: 10.1097/MD.0000000000051483 (PMC13747548; doi:10.1097/MD.0000000000051483)
Supplement: Supplementary file 1 [file medi-105-e51483-s001.docx]

**Table S1, Supplemental Digital Content. Thyroid hormone levels in the proband and his mother**

| **Individual** | **FT3 (pmol/L)** | **FT4 (pmol/L)** | **Total T3 (nmol/L)** | **Total T4 (nmol/L)** | **TSH (mIU/L)** |
| --- | --- | --- | --- | --- | --- |
| **Proband** | 6.35 H (2.63–5.70) | 6.01 L (9.01–19.03) | 4.70 H (1.07–2.78) | 40.60 L (65.9–135.2) | 6.83 H (0.35–4.94) |
| **Mother** | 5.68 (2.63–5.70) | 10.2 (9.01–19.03) | 2.91 H (1.07–2.78) | 92.4 (65.9–135.2) | 2.31 (0.35–4.94) |

**Abbreviations:** FT3, free triiodothyronine; FT4, free thyroxine; T3, triiodothyronine; T4, thyroxine; TSH, thyroid-stimulating hormone. Values in parentheses are laboratory reference intervals. H, above the upper reference limit; L, below the lower reference limit.
