# Supplementary material for: Allan-Herndon-Dudley syndrome associated with SLC16A2 c.1384G>A (p.Gly462Arg): A family case report with retrospective molecular diagnosis in a deceased sibling
Source: Medicine (Baltimore). 2026 Nov 6;105(45):e51483. doi: 10.1097/MD.0000000000051483 (PMC13747548; doi:10.1097/MD.0000000000051483)
Supplement: Supplementary file 2 [file medi-105-e51483-s002.docx]

**Table S2, Supplemental Digital Content. Pathogenicity assessment of *SLC16A2* c.1384G>A (p.Gly462Arg)**

| **Evidence** | **Details** | **Criterion** | **Strength** |
| --- | --- | --- | --- |
| **Population data** | Absent from gnomAD versions queried at testing, ExAC, 1000 Genomes, and the laboratory’s local East-Asian exome set (>15,000 exomes). | PM2_Supporting | Supporting |
| **Computational evidence** | PolyPhen-2: probably damaging (1.000); SIFT: deleterious (0.00); CADD: 29.6; REVEL: 0.932; MutationTaster: disease causing; Gly462 conserved to zebrafish (GERP++ RS 5.78). REVEL 0.932 meets a ClinGen-calibrated threshold for strong pathogenic computational evidence. | PP3_Strong | Strong |
| **Phenotype** | Severe AHDS phenotype with the characteristic high-T3 / low-T4 profile of MCT8 deficiency. | PP4 | Supporting |
| **Segregation** | Same hemizygous variant in the similarly affected maternal half-brother; mother heterozygous. | PP1 | Supporting |
| **Final classification** | PM2_Supporting + PP3_Strong + PP4 + PP1 (ACMG/AMP with calibrated PP3). | Likely pathogenic | — |

**Abbreviations:** ACMG, American College of Medical Genetics and Genomics; AHDS, Allan-Herndon-Dudley syndrome; AMP, Association for Molecular Pathology; CADD, Combined Annotation Dependent Depletion; ClinGen, Clinical Genome Resource; GERP++, Genomic Evolutionary Rate Profiling; MCT8, monocarboxylate transporter 8; REVEL, Rare Exome Variant Ensemble Learner; RS, rejected substitutions; SIFT, Sorting Intolerant From Tolerant. Computational predictors are counted once under PP3. Helix 11 topology and the 2017 published case were used as context only and were not assigned separate ACMG codes.
